# Diurnal patterns of sedentary behavior and changes in physical function over time among older women: a prospective cohort study

**DOI:** 10.1186/s12966-020-00992-x

**Published:** 2020-07-09

**Authors:** Chase Reuter, John Bellettiere, Sandy Liles, Chongzhi Di, Dorothy D. Sears, Michael J. LaMonte, Marcia L. Stefanick, Andrea Z. LaCroix, Loki Natarajan

**Affiliations:** 1 Department of Family Medicine and Public Health, University of California San Diego, San Diego, California 92093 USA; 2 Center for Behavioral Epidemiology and Community Health (CBEACH), San Diego State University, San Diego, CA 92123 USA; 3 Division of Public Health Sciences, Fred Hutchinson Cancer Research Center, Seattle, WA 98109 USA; 4 College of Health Solutions, Arizona State University, Phoenix, AZ 85004 USA; 5 Moores Cancer Center, University of California San Diego, 3855 Health Sciences Dr, La Jolla, CA 92037 USA; 6 Department of Epidemiology and Environmental Health, School of Public Health and Health Professions, University at Buffalo–SUNY, New York, NY 14214 USA; 7 Stanford Prevention Research Center, Stanford University School of Medicine, Stanford University, Stanford, CA 94305 USA

**Keywords:** Sedentary behavior, Physical functioning, Older adults, Clustering, K-means, Hierarchical, Circadian

## Abstract

**Background:**

Sedentary behavior (SB) is linked to negative health outcomes in older adults. Most studies use summary values, e.g., total sedentary minutes/day. Diurnal timing of SB accumulation may further elucidate SB-health associations.

**Methods:**

Six thousand two hundred four US women (mean age = 79 ± 7; 50% White, 34% African-American) wore accelerometers for 7-days at baseline, yielding 41,356 person-days with > 600 min/day of data. Annual follow-up assessments of health, including physical functioning, were collected from participants for 6 years. A novel two-phase clustering procedure discriminated participants’ diurnal SB patterns: phase I grouped day-level SB trajectories using longitudinal k-means; phase II determined diurnal SB patterns based on proportion of phase I trajectories using hierarchical clustering. Mixed models tested associations between SB patterns and longitudinal physical functioning, adjusted for covariates including total sedentary time. Effect modification by moderate-vigorous-physical activity (MVPA) was tested.

**Results:**

Four diurnal SB patterns were identified: p1 = high-SB-throughout-the-day; p2 = moderate-SB-with-lower-morning-SB; p3 = moderate-SB-with-higher-morning-SB; p4 = low-SB-throughout-the-day. High MVPA mitigated physical functioning decline and correlated with better baseline and 6-year trajectory of physical functioning across patterns. In low MVPA, p2 had worse 6-year physical functioning decline compared to p1 and p4. In high MVPA, p2 had similar 6-year physical functioning decline compared to p1, p3, and p4.

**Conclusions:**

In a large cohort of older women, diurnal SB patterns were associated with rates of physical functioning decline, independent of total sedentary time. In particular, we identified a specific diurnal SB subtype defined by less SB earlier and more SB later in the day, which had the steepest decline in physical functioning among participants with low baseline MVPA. Thus, diurnal timing of SB, complementary to total sedentary time and MVPA, may offer additional insights into associations between SB and physical health, and provide physicians with early warning of patients at high-risk of physical function decline.

## Background

With accelerated growth of the older adult population [1], identifying ways to prevent disease and promote healthy aging is becoming a major public health focus. Older women have proportionately higher morbidity and disability than older men [2], despite having a longevity advantage. Because of this, women have more hospitalizations and outpatient visits, longer utilization of long-term care services, and greater health care spending [3–5]. These disparities make the identification of modifiable risk factors for declining health especially important for older women.

A growing body of recent research suggests that a promising risk factor to target is sedentary behavior (SB). In the United States, older adults are estimated to spend an average of 8 to 9.5 h per day in SB [6–8]. Older adults accumulate the greatest volume of sedentary time of any age group [9–12], and older age is associated with more sedentary time even among older adults [13]. In older adults, SB has been linked with higher risk of cardiovascular disease, metabolic syndrome, diabetes, and all-cause mortality [14–16].

Accelerometers have been increasing our understanding of SB and how it relates to health. Early studies used self-reported measurements of SB, which can be unreliable or inaccurate, especially in older age groups [17–19]. Accelerometer data are subject to less measurement error, and analyses of these data have revealed stronger associations between SB and health than were previously observed using self-reports [20–22].

Accelerometers also enable researchers to study diurnal patterns of sedentary time [23]. Studies analyzing objectively measured sedentary time data usually reduce them to a single measure—total minutes per day spent sedentary—overlooking diurnal accumulation patterns that may yield important information about health outcomes. While it is recognized that high levels of SB are negatively related to health, the association may vary depending on when sedentary time is accumulated during the waking day.

While there is a paucity of evidence relating diurnal patterns of SB with health, emerging evidence suggests that diurnal patterns of physical activity (PA) are associated with fatigability [24]; early-stage Alzheimer’s disease [25]; insulin and C-reactive protein levels, and quality of life [26]; sleep efficiency, cognition, and all-cause mortality [27]. There have also been reports that diurnal patterns of SB vary by sociodemographic variables; for example, older adults accumulate more SB during the day than younger adults, with smaller age group differences in evening SB accumulation [28]. These data support the need to study diurnal patterns of SB to determine possible associations with health-related outcomes.

Using a novel, two-stage clustering procedure, we investigated diurnal patterns of SB and how these were related to trajectories of physical functioning. Data were drawn from a study in which 6489 women wore ActiGraph accelerometers on their waist for up to 7 days at baseline, and then were followed for up to 6 years with annual assessments of physical functioning [8]. We expected diurnal patterns of SB to be associated with trajectories of physical functioning among this cohort of community-living older women.

## Methods

### Sample and design

Data were used from participants in the Objective Physical Activity and Cardiovascular Health Study (OPACH), a subset of Women’s Health Initiative participants who had enrolled in the Long Life Study. Women’s Health Initiative women in the Long Life Study consented to periodic in-home examinations to provide blood samples, updated health information, and physical measurements. More details of the ancillary OPACH study, which was designed specifically for collection of objective measures of physical behavior, have been previously published [8]. In brief, the 7048 ambulatory, community-dwelling women who consented to participate in OPACH were given ActiGraph GT3X+ accelerometers on a waist belt and asked to wear them 24 h per day (except when bathing or swimming) for 7 consecutive days. They concurrently recorded in-bed and out-of-bed times each day in sleep logs. Accelerometers were returned by 95.4% (6721) of participating women and 92.1% (6489) contained evidence of human wear [29]. Each participant was followed for up to 6 years with annual medical updates collecting information on physical functioning.

### Outcome: physical functioning

Physical functioning was assessed yearly using the RAND 36 Health Survey physical function subscale, a 10-item, well-validated self-report measure [30, 31]. The physical functioning scale assesses current health limitations on physical functioning during daily activities. Item scores are averaged, producing a score from 0 to 100, with higher values indicating superior physical functioning. Median number of physical functioning assessments available (25th %, 75th %) was 5 (4, 5) time points.

### Accelerometer measurement

Raw accelerometer data (30 hertz) were converted using ActiLife software (Version 6) to vector magnitude counts per 15-s epochs. To remove accelerometer non-wear time, we applied the Choi algorithm (90-min window, 30-min stream frame, and 2-min tolerance) to the minute-level vector magnitude counts [32] and identified in-bed time using data reported in the sleep logs. Missing bed times were imputed using the mean in-bed and out-of-bed time of each person when available; for the 434 participants who had in-bed and/or out-of-bed data missing in all days, the mean in-bed (10:45 pm) and/or out-of-bed (7:22 am) time for the population was used. We defined adherent days as calendar days with 10 h or more of awake wear time [33], and only analyzed adherent days. Because our metrics of sedentary time and sedentary accumulation patterns were intended to estimate typical behavior during a week, at least 4 adherent days were required for inclusion of a participant’s data in analyses [33]. Out of the 6489 women with wear time, 95.6% (6204) had at least 4 adherent days.

Accelerometer data were processed using intensity-specific cutpoints that had been developed specifically for the OPACH cohort in a laboratory calibration study of 200 women aged 60–91 years who wore ActiGraph GT3X+ accelerometers on their hip [34]. Sedentary time was classified as any 15-s epoch at or below 18 vector magnitude counts and MVPA was any 15-s epoch above 519 vector magnitude counts.

### Covariates

Questionnaire data collected at Women’s Health Initiative baseline included age, race/ethnicity (Black, White, or Hispanic) and education (high school graduate/general education development (GED) or less, some college, college graduate/advanced degree). Participants completed questionnaires at OPACH baseline that included measures of self-reported health (excellent/very good, good, fair/poor), frequency of alcohol consumption (non-drinker, less than one drink/week, one or more drinks/week, unknown), and current smoking status (smoker vs nonsmoker). Measures of weight and height were taken during Long Life Study in-home visits using a calibrated analog scale and a tape measure, and used to compute body mass index (BMI) as weight (kilograms)/height (meters^2^). Morbidity was measured at OPACH baseline as the number of the following chronic health conditions: cardiovascular disease; cancer; osteoarthritis; cognitive impairment; depression; history of falls over the previous 12 months; cerebrovascular disease; chronic obstructive pulmonary disease; diabetes; vision impairment; hearing loss [35].

### Statistical methods

#### Clustering procedure

Sedentary minutes were summed for each one-hour interval of the day (e.g., 6:00 am-6:59 am, 7:00 am – 7:59 am) to describe hourly behavior. Inclusive of the first hour that participants were awake and wore the accelerometer for 60 min, daily trajectories of sedentary behavior were obtained using data from 14 consecutive hours. This consistent duration across days was required to implement the longitudinal clustering, and 14 h was selected based on the degree of missingness (i.e., 23.6% of data from the 14th hour was missing while 49.5% of data from the 15th hour was missing). For hours with missing data, sedentary time was imputed based on linear interpolation with added stochastic variation based on population trajectories within missing time points (a method known as “Copy Mean”) [36]. An alternative method for computing trajectories would be to use a fixed range of clock time (e.g. 8 am to 10 pm) instead of beginning with participants’ wake time. That alternative method, while a reasonable approach to obtaining SB diurnal patterns, does not conform with our understanding that participants’ diurnal patterns commence with their highly variable time of awakening rather than at a fixed hour of the day. For our primary analyses, we adopted the method of beginning the day at the participant’s hour of awakening, but conducted a concordance analysis using the alternative method (i.e., using clock-time to define start-of-day).

A novel two-phase clustering procedure was then used to group OPACH participants according to their diurnal and day-to-day variability in SB patterns. In Phase I, k-means for longitudinal data (*kml*) was conducted on all available days of data from the entire sample (n_days_ = 41,356) to determine clusters of *days* that optimally differentiated diurnal trajectories. To select the number of clusters in Phase I, we used the Calinski-Harabasz criterion [37]. To address cluster stability, the kml algorithm was repeated five times with different starting conditions, and the partition that led to the highest Calinski-Harabasz criterion was selected. In Phase II, hierarchical clustering with complete linkage [38] was conducted on the proportion of a participant’s measurement days assigned to each of the identified day clusters, to determine the diurnal SB patterns of participants. The proportion of days was used because the number of days available for each participant varied from 4 to 7 days. To determine the number of SB pattern clusters, we selected the solution that maximized average silhouette width across observations [39]. Thus, Phase I clustered days based on similarities in within-day timing of SB accumulations, whereas Phase II clustered individuals based on similarities in their between-day SB patterns, e.g., how they distribute days within a week into various SB accumulation clusters.

#### Descriptive statistics

Socio-demographic, health-related, and activity-related variables were summarized for each of the groups of participants clustered according to their diurnal SB patterns, using means and standard deviations (SD) for continuous variables and percentages for categorical variables with differences tested using F-tests and chi-square tests, respectively.

#### Mixed-effects regression

Multivariable linear mixed-effects regression tested whether diurnal SB patterns were associated with trajectories of physical functioning. This enabled investigation of differences in baseline physical functioning by diurnal SB pattern (aka, the y-intercept of the trajectory), and how the slopes of the physical functioning trajectories changed over time in relation to these patterns. The general model was physical functioning_it_ = pattern_i_ + time_it_ + pattern_i_*time_it_ + covariates_i_, with subjects i = 1,…,N, and year of follow-up t = 1,…,T. A person-level random intercept was also included in the model to account for within-person correlations. Model covariates included age, race-ethnicity, BMI, education, smoking status, alcohol use, number of morbidities, and self-rated health. To test whether associations of diurnal SB patterns with trajectories of physical functioning reflected daily sedentary time, we additionally adjusted for total sedentary time. Based on previous literature, we examined whether associations of diurnal SB patterns and physical functioning varied by MVPA (which, as in our previous studies [15, 40], has been modeled as a binary variable using a median split to increase statistical power within each MVPA subgroup and because there is no older-adult-specific threshold that has been established using accelerometer data) [40]. This was accomplished by adding a pattern*time*MVPA interaction and using a likelihood ratio test to assess statistical significance. The potential influence of using complete case analysis was assessed by using multivariate imputation by chained equations [41] with predictive mean matching and Bayesian polytomous regression for continuous and categorical variables, respectively.

All analyses were conducted in R (version 3.5.3), including the use of packages *kml* [42], *cluster* [39], *nlme* [43], and *mice*. All tests were two-tailed with alpha = 0.05 unless otherwise specified. Sample R code for our cluster analysis is available at https://github.com/Aging-and-Behavioral-Epidemiology/two_stage_clustering.

## Results

### Study sample and baseline descriptive characteristics

Our analytic sample comprised 6204 OPACH participants, with mean (SD) age of 79 (7) years at study entry, 49.6% White, 33.6% Black, 41.0% completed college or higher. Mean (SD) BMI at baseline was 28.1 (5.7). 2.6% of participants were current smokers, and 25.8% had 1 or more alcoholic drinks per week in past 3 months before baseline. 89.2% of participants rated their general health as excellent, very good, or good. 17.5, 34, 26.8, and 21.6% had no, 1, 2, or 3 or more morbidities at baseline, respectively. Participants wore accelerometers for a mean (SD) 6.9 (0.3) days, yielding 41,356 days with mean (SD) 14.9 (1.3) hours/day of data. At study entry, mean (SD) daily sedentary time and MVPA were respectively 597 (103), and 50 (34) minutes. Mean (SD) physical functioning scores at baseline were 69 (26).

### Diurnal SB patterns defined by the two-phase clustering procedure

**Fig. 1:**
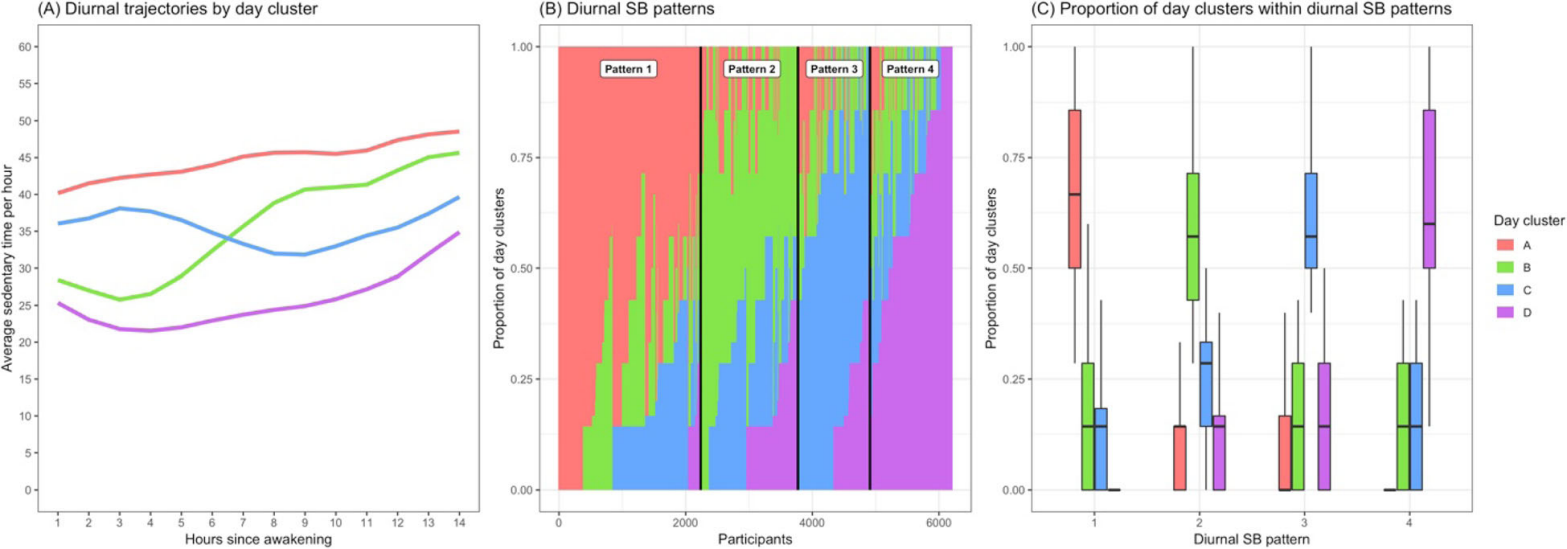
Derivation of the diurnal SB pattern exposure variable. This panel displays day clusters of diurnal trajectories and their distribution at the individual participant level and the diurnal SB pattern level. Panel (**a**) displays the diurnal trajectory of each day cluster as defined by the average sedentary time across days within respective clusters. Panel (**b**) shows the distribution of day clusters for each participant, i.e. each point on the x-axis represents a participant. Diurnal SB pattern boundaries are also marked. Panel (**c**) shows boxplot distributions of day cluster proportions within diurnal SB patterns

In Phase I, k-means for longitudinal data (*kml*) was conducted on all available days of data (n_days_ = 41,356) to select the optimal clusters of days. We found a solution of four clusters that had a sufficiently high Calinski-Harabasz criterion [37], yet with enough variability among clusters to clearly differentiate diurnal trajectories; the chosen partition also had the lowest AIC and BIC. Based on visual inspection, the four day clusters can be described as: (A) an overall high level of SB throughout the day, (B) a moderate level of SB, with a lower level of SB at start of day, relative to the end of day, (C) a moderate level of SB, with a higher level of SB at the start of day, relative to the end of day, and (D) an overall low level of SB throughout the day (Fig. 1a). Of note, clusters A, B, and D showed increasing SB trends over the course of the day, whereas cluster C had higher SB earlier followed by ~ 6 h of a decreasing trend, and then increasing for the rest of the day.

Then in Phase II, hierarchical clustering was conducted on the proportion of each participant’s days that fell within each of the four Phase I day clusters. We found that a 4-cluster solution maximized average silhouette width across observations [39]. Diurnal SB patterns 1, 2, 3, and 4 can be described according to which day cluster A, B, C, and D, respectively, their days predominantly fell into (see Fig. 1 b, c). For example, for individuals in diurnal SB pattern 1 the median (25th%, 75th%) proportions of cluster A (high SB) days were 66.7% (50, 85.7%) vs 0% (0, 0%) proportion of cluster D (low SB) days. Conversely, for SB pattern 4 the median (25th%, 75th%) proportions were 0% (0, 0%) vs 60.0% (50.0, 85.7%) for cluster A and cluster D days, respectively. SB patterns 2 and 3 had similar distributions of high SB (cluster A) and low SB (cluster D) days, but differed according to the proportions of cluster B vs C days: for SB pattern 2 the median (25th%, 75th%) proportions for cluster B vs C days were 57.1% (42.9, 71.4%) vs 28.6% (14.3, 33.3%), respectively, whereas SB pattern 3 had 14.3% (0, 28.6%) vs 57.1% (50.0, 71.4%), respectively. Thus, on average, SB patterns 1–4 were distinguished by the corresponding day clusters A–D, of which each pattern was primarily composed.

### Concordance analysis for the clustering procedure

Of two alternative accelerometer processing procedures on which to base our clustering analyses, we chose the procedure using data aligned to the per subject start of day rather than data aligned to clock time of day. Reassuringly, when we conducted a concordance analysis to compare the procedures, findings were similar: the two procedures resulted in 81.4% of the days being classified in the same day cluster, and 73.2% of the participants being classified in the same diurnal SB pattern.

### Descriptive statistics by diurnal SB pattern

Baseline participant characteristics by SB pattern are in Table 1. The low SB pattern [4] and high SB pattern (1) had characteristics that correlated with better and worse health, respectively. For example, those with the high SB pattern had on average higher age, BMI, proportion of current smokers, number of morbidities, total sedentary time; they also had lower self-rated health, MVPA time, and physical functioning. Both moderate SB patterns (2 and 3) were on average similar on most characteristics, with values closer to the sample averages.

**Table 1:** Baseline demographic, lifestyle, health indicators and physical functioning by diurnal SB pattern

Variable	Diurnal SB Pattern								***p***-value
	1 (***n*** = 2239)		2 (***n*** = 1536)		3 (***n*** = 1137)		4 (***n*** = 1292)		
Age (years), mean (SD)	80.6	(6.5)	78.4	(6.4)	78.1	(6.8)	76.3	(6.3)	< 0.001
Ethnicity, n (%)									< 0.001
White	1316	(58.8)	752	(49)	502	(44.2)	506	(39.2)	
Black	699	(31.2)	478	(31.1)	459	(40.4)	449	(34.8)	
Hispanic	224	(10.0)	306	(19.9)	176	(15.5)	337	(26.1)	
Education, n (%)									< 0.001
HS/GED or less	434	(19.5)	337	(22.1)	199	(17.7)	281	(21.8)	
Some college	936	(42.1)	567	(37.2)	407	(36.2)	477	(37.0)	
College grad or more	853	(38.4)	622	(40.8)	519	(46.1)	531	(41.2)	
BMI (kg/m^2^), mean (SD)	29.7	(6.1)	27.5	(5.3)	27.7	(5.4)	26.5	(5.1)	< 0.001
Current smoker (Yes), n (%)	73	(3.3)	37	(2.4)	32	(2.8)	20	(1.5)	0.02
Alcohol in past 3 months, n (%)									< 0.001
0 drinks per week	858	(38.3)	471	(30.7)	382	(33.6)	415	(32.1)	
< 1 drinks per week	690	(30.8)	538	(35.0)	331	(29.1)	371	(28.7)	
≥ 1 drinks per week	460	(20.5)	424	(27.6)	309	(27.2)	408	(31.6)	
Unknown	231	(10.3)	103	(6.7)	115	(10.1)	98	(7.6)	
Number of morbidities^a^, n (%)									< 0.001
0	298	(13.4)	293	(19.2)	197	(17.4)	295	(22.9)	
1	671	(30.1)	567	(37.1)	380	(33.6)	482	(37.4)	
2	623	(27.9)	401	(26.3)	318	(28.1)	315	(24.5)	
≥ 3	637	(28.6)	266	(17.4)	237	(20.9)	196	(15.2)	
Arthritis (Yes), n (%)	1262	(56.4)	822	(53.5)	638	(56.1)	683	(52.9)	0.11
Diabetes (Yes), n (%)	546	(24.4)	285	(18.6)	245	(21.5)	198	(15.3)	< 0.001
Stroke (Yes), n (%)	134	(6.0)	63	(4.1)	49	(4.3)	39	(3.0)	< 0.001
Self-rated general health, n (%)									< 0.001
Excellent or very good	909	(40.7)	842	(55.0)	588	(51.9)	777	(60.4)	
Good	1017	(45.6)	576	(37.6)	441	(38.9)	424	(33.0)	
Fair or poor	306	(13.7)	114	(7.4)	104	(9.2)	85	(6.6)	
Sedentary time (minutes/day), mean (SD)	668.8	(85.0)	577.5	(75.7)	592.7	(78.0)	501.2	(86.9)	< 0.001
MVPA (minutes/day), mean (SD)	28.6	(19.2)	54.2	(28.8)	50.7	(28.0)	82.1	(38.9)	< 0.001
Wear time (hours/day), mean (SD)	14.7	(1.4)	14.8	(1.3)	15.1	(1.3)	15.0	(1.3)	< 0.001
Wear days, mean (SD)	6.4	(0.9)	6.5	(0.8)	6.5	(0.9)	6.5	(0.8)	0.03
Physical functioning, mean (SD)	58.2	(27.5)	73.7	(22.8)	71	(24.1)	79.3	(21.5)	< 0.001

Group differences are tested using ANOVA for continuous variables and chi-squared tests for discrete variables

*Abbreviations*: *SD* standard deviation, *HS* high school, *GED* general educational development, *BMI* body mass index, *MVPA* moderate-to-vigorous physical activity

^a^cardiovascular disease, cancer, osteoarthritis, cognitive impairment, depression, history of falls over the last 12 months, cerebrovascular disease, chronic obstructive pulmonary disease, diabetes, vision impairment, hearing loss

### Mixed-effects regression: associations between physical functioning and diurnal SB patterns

**Fig. 2:**
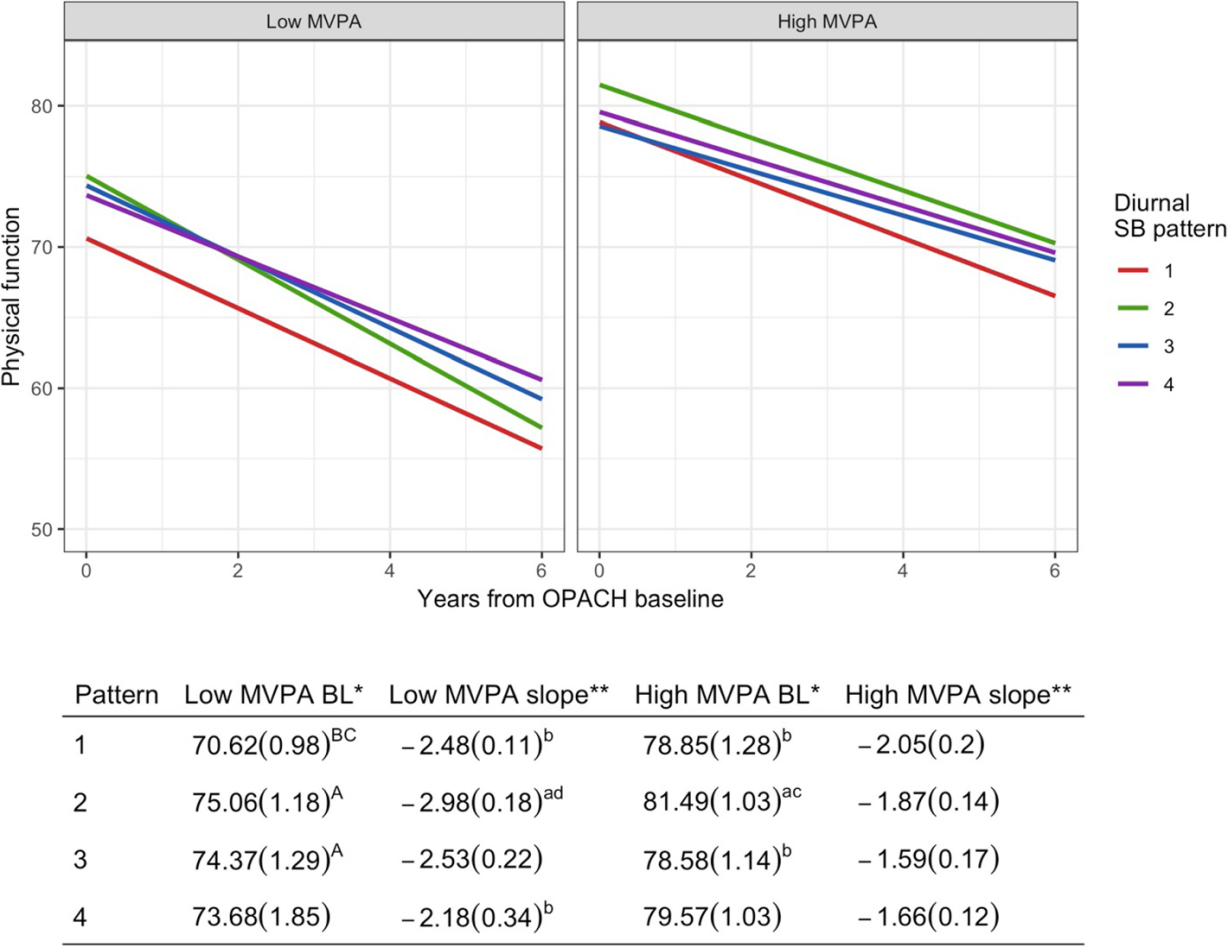
Baseline and slope of physical functioning by diurnal SB pattern and high and low MVPA. The multivariable model is adjusted for age, race-ethnicity, body mass index (BMI), education, smoking status, alcohol use, number of morbidities, self-rated health, and accelerometer-measured total sedentary time. BL and slope estimates (standard error) of PF are derived from 3-way interaction model described above. MVPA is dichotomized into “high” and “low” according to the median value (43.21 min/day). Abbreviations: BL = baseline; MVPA = moderate-to-vigorous physical activity. *All patterns had significant differences, *p* < 0.01 between their respective high and low MVPA baseline values. **Patterns 2 and 3 had significant differences, p < 0.01, between their respective high and low MVPA slopes. ^abcd^Lowercase a,b,c,d indicate a significant difference, *p* < 0.05, with the BL or slope of pattern 1,2,3,4, respectively. ^ABCD^Uppercase A,B,C,D indicate a significant difference, p < 0.01, with the BL or slope of pattern 1,2,3,4, respectively

In the study sample, mean physical functioning decline was 2.2 points/year (standard error = 0.07). MVPA was a significant modifier of the association between diurnal SB pattern and physical functioning (likelihood ratio *p* < 0.0001), and therefore results are presented stratified by high and low MVPA (Fig. 2). Baseline physical functioning levels were significantly higher for women with high MVPA (ranging from 78.6 to 81.5) than for women with low MVPA (range from 70.6 to 75.1). Women in all SB patterns experienced decline in physical functioning over time. The decline in physical functioning over time was significantly slower in SB pattern 2 and 3 women with high MVPA (1.9 and 1.6 points/year, respectively) than the physical functioning decline among women in the same SB pattern with low MVPA (3.0 and 2.5 points/year, respectively).

Among women with low MVPA, those in the high SB pattern [1] had lower baseline physical functioning (70.6 ± 1.0) than all other patterns, which was significantly lower than both of the moderate SB patterns (pattern 2 physical functioning score = 75.1 ± 1.2; pattern 3 physical functioning score = 74.4 ± 1.3). The pattern having lower SB at the start of the day relative to the end of day (pattern 2) had the steepest negative slope of physical functioning decline (− 3.0 ± 0.2), which was significantly steeper than that of the consistently low SB pattern [4] and consistently high SB pattern [1]. There were no significant differences in baseline physical functioning levels or changes in physical functioning over time between the two moderate diurnal SB patterns.

Among women with high MVPA, the women in SB pattern 2 had significantly higher baseline physical functioning (81.5 ± 1.0) than those in SB patterns 1 and 3 (78.9 ± 1.3, and 78.6 ± 1.1, respectively). The women in the consistently high SB pattern [1] had the most rapid decline in physical functioning over time, though not significantly different than that of the other diurnal SB pattern groups. The rate of physical functioning decline among women with low morning and high evening SB pattern [2] was not significantly different than that in the most rapidly declining group (pattern 1; *p* = 0.25). Women with a consistently low diurnal SB pattern [4] and women with a high morning and low evening SB pattern [3] had slightly slower declines in physical functioning (1.7 ± 0.1 and 1.6 ± 0.2 physical functioning points per year) than those in the persistently high SB pattern (1; 2.1 ± 0.2 physical functioning points per year; respective *p*-values = 0.10 and 0.08). Results were similar with and without adjustment for total sedentary time (Additional File 1).

Similar results were obtained with and without multiple imputation, likely due to the relatively small amount of missingness (records removed due to listwise deletion = 1796/27717, or 6.5%). As a result, all analyses were conducted using complete cases.

## Discussion

Summary measures of sedentary behavior, e.g., daily sedentary minutes, are linked to negative health in older adults. By leveraging minute-level accelerometry, we derived clusters of diurnal timing of sedentary behavior to further elucidate associations with health. We used a two-phase clustering approach that first elicited daily (hour-by-hour) trajectories of SB time accumulation, and then clustered individuals based on their daily patterns across multiple days. This approach allowed us to group participants according to the amount of daily SB, as well as the timing of their SB accumulation. E.g., we identified two clusters that had similar (moderate) total daily SB yet differed in the phase of the day (morning vs evening) wherein most sedentary time was accumulated. Such delineation is not possible if only total daily SB is used to characterize an individual’s SB.

We next investigated whether the derived diurnal SB patterns were associated with cross-sectional and longitudinal markers of health. Unsurprisingly, we found that higher sedentary behavior throughout the day was generally associated with poorer health indicators (high BMI, lower physical functioning, more morbidities) compared to moderate or lower sedentary behavior. However, a novel and intriguing finding was that among participants who had below median MVPA with moderate daily sedentary behavior, those who were less sedentary earlier in their day (vs later) had significantly higher physical functioning scores at study entry but also steeper decline in longitudinal physical health compared to those who had high levels of sedentary behavior throughout the day. Thus, we seem to have identified a latent subgroup that is at high risk of physical functioning decline, a finding that would have been masked had we only used total daily SB as the exposure of interest. Interestingly, these effects were not as apparent in women who had above-median levels of MVPA. In particular, although patterns 1 and 2 exhibited higher rates of decline than patterns 3 and 4, the effect-sizes were smaller, suggesting that MVPA may partially mitigate the negative effects of diurnal sedentary behavior patterns. This finding is similar to recently-published results showing that self-reported sitting time is more strongly related to all-cause mortality among adults with low levels of MVPA compared to adults with higher levels of MVPA [44, 45].

While to our knowledge this is the first study to evaluate diurnal patterns of SB in relation to physical function, Schrack et al. observed trends in physical activity accumulation across the day, showing that morning PA was on average similar across age groups for the 611 adults (aged 32–93) they studied, but that older adults had significantly less activity as the day progressed than younger adults [46]. They hypothesized that, “Older adults tended to reach their peak activity level much earlier in the day, which may indicate that daily tasks such as bathing, dressing, running errands, and volitional physical exercise are performed in the morning hours with little activity later in the day.” Our data expand on this work by showing that even among a population consisting solely of older adults and after accounting for their chronological age, there is noticeable diurnal variation in accumulation of SB. Furthermore, compared to consistently high SB throughout the day (i.e., cluster p1), we found that a pattern with lower SB earlier and higher SB later in the day (i.e., cluster p2) is related to accelerated functional decline, among participants who engaged in low MVPA. The extent to which this novel observation represents a causal relationship that evening accumulation of SB is more deleterious to physical function than morning SB, requires further study. It is possible that there is an underlying biological ageing process that affects both diurnal SB patterns and trajectories of physical function. Or, sedentary behavior later in the day may disrupt or change circadian rhythms of biological processes linked to ageing and chronic disease. It is important to note that the high-risk cluster (p2) in fact generally had better health (i.e., fewer morbidities, lower BMI) at baseline relative to the high-SB (p1) group. If confirmed in other studies, knowing that specific diurnal activity patterns are associated with increased risk for accelerated decline might identify high-risk patients, who might not otherwise be considered high risk, and alert clinicians early to these patients. In the future, this might trigger a suite of interventions to prevent such decline.

In our older adult population, multiple health conditions are prevalent, which could confound the observed associations. We attempted to adjust for these health conditions by including a “number of morbidities” covariate. However, it is possible that specific morbidities may exert stronger confounding effects. To this end, we conducted a sensitivity analysis in which we controlled for specific conditions, such as stroke, diabetes, and arthritis, which would be strongly related to SB and physical function. The results of this sensitivity analysis were similar to our original findings (Additional File 2). Another important confounding factor is vision loss and the likely higher fear of falling among participants with vision loss [47]. We emphasize that while our analysis adjusted for a host of measured confounders, we cannot rule out residual confounding and acknowledge this as a limitation.

We used physical functioning trajectories across 6 years as a measure of overall functioning and health in our population of older women. The validated RAND-36 physical functioning subscale is reported to be a comprehensive marker of mobility-disability among older adults [48]. It is recognized that aging-related decline in health is a multidimensional phenomenon [49, 50], and the physical functioning score used herein may be regarded as an overarching health indicator that captures multiple facets and morbidities of health decline in older adults. A natural next step would be to identfy associations between diurnal SB patterns and specific biological and self-reported outcomes that may underlie the current finding. We leave this to future research.

Many investigators have developed new analytic approaches for accelerometer data in an effort to not just measure total accumulation of PA/SB time but to detect *patterns* in the data. Several studies have investigated patterns based on lengths of uninterrupted intervals (“bouts”) and/or degree of fragmentation of PA/SB [15, 22, 51–57]. More relevant to the current investigation, studies have also described variations in the *timing* of sedentary time and PA time accumulation with respect to hour of the day and/or day of the week, including differences in timing by sociodemographic factors [23, 28, 58–62]. None of these studies about the timing of PA/SB attempted to determine whether the identified patterns were associated with health status. We identified a few studies that examined *diurnal* patterns of PA/SB, (i.e., patterns based on the time of day that active/sedentary time was accumulated) and health. Using functional principal components analysis, Xu et al. reported that higher evening (vs mid-day) activity was associated with worse mental quality-of-life [26]. Zeitzer et al. found principal components-derived patterns that were predictive of changes in sleep and cognition, as well as cardiovascular-related mortality and all-cause mortality [63]. However, both studies modeled accelerometer/ActiGraph counts as the exposure measure of activity and did not examine SB directly. Also, the study design used by Xu et al. was cross-sectional, and they did not investigate longitudinal trajectories of quality-of-life. In another study, using k-means analysis of METs data (metabolic equivalent of tasks), Fukuoka et al. discriminated three groups of participants according to the times of day at which their peak levels of MVPA occurred [63]. The MVPA evening peak group had significantly higher BMI, waist circumference, and hip circumference than the MVPA noon peak group. An x-means cluster analysis study of diurnal patterns, similar to our own, identified 4 distinct PA clusters on the basis of intensity and temporal patterns of activity, and found that risk of cardiovascular disease varied across clusters in both males and females [64]. The functional principal components analysis approach discerns the major modes of variation in PA/SB patterns, while our and other clustering methods identify distinct homogeneous subgroups of participants. Fukuoka et al. and Niemala et al. derived clusters using diurnal patterns of overall activity measured via METs, whereas our approach focuses specifically on SB. For behavioral intervention studies, eliciting timing of specific behaviors (such as SB or MVPA) may be most useful for tailoring interventions to the needs of the individual. For instance, given findings from our study, a behavioral intervention aiming to reduce SB would offer different strategies to individuals who have one versus the other of the two moderate SB patterns.

Our study has many strengths. Our sample comprised a large diverse and well-characterized cohort of > 6000 older participants from the Women’s Health Initiative. The rich Women’s Health Initiative database contains detailed data on a large number of variables, allowing us to develop robust models and adjust for a number of potential confounders. We used objective accelerometers to measure SB and PA, and leveraged minute-level assessments to ascertain diurnal variation in SB patterns. Our 2-stage clustering approach uncovered latent subgroups based on within-day and between-day similarity in SB patterns, which could be useful for developing targeted interventions to reduce SB, e.g., when during the day to intervene (morning vs afternoon) and which day (e.g., a high SB-day). Due to the availability of multiple longitudinal measures of physical functioning, we were able to examine how SB pattern impacts not only current physical functioning but also future rate of decline. There are also limitations. Clustering analysis is an unsupervised, inherently exploratory technique. While we conducted a variety of sensitivity analyses to assess the stability of our results, additional independent studies are needed to replicate (or refute) our findings. Also, the derived clusters in our study provide broad groupings of similar SB patterns. Although finer-grained groupings are possible, we elected to maintain a coarser classification, so as not to overfit the data at hand. If our findings are replicated, further subdivisions of clusters may offer additional insights. In our study, we treated weekday and weekend days equally. While studies among similarly aged older adults indicate that total sedentary time does not significantly vary by weekday vs weekend day [65, 66], there is evidence that diurnal accumulation patterns may differ and future studies should further explore this to guide development of personalized interventions. Our physical functioning measure, albeit validated and used extensively in “aging” research, is self-reported. Replicating our results using longitudinal objective outcomes of physical health would strengthen our findings. Even though we were able to adjust for a large number of covariates, unmeasured confounding could still be present (e.g., by variables such as occupation that were not measured in OPACH), and must be acknowledged when interpreting our results. E.g., the finding that the moderate-SB-with-lower-morning-SB pattern [2] had steeper physical functioning decline could signify a latent subgroup at high risk of decline, but could also be due to an unmeasured baseline factor. Similarly, in our observational study it is not possible to infer the direction of associations between MVPA (or other baseline factors) and baseline physical functioning. Even so, availability of longitudinal measures of physical functioning allows us to infer effects of baseline MVPA on future physical functioning decline, thus partially addressing this concern. Further investigation of the sequence of cause and effects is warranted.

## Conclusions

In summary, in a large cohort of older, ambulatory, community-living women, we found that diurnal SB patterns were associated with differential rates of physical functioning decline. Diurnal timing of SB, complementary to total sedentary time and MVPA, may offer additional insights into associations between SB and physical health.

## Supplementary information

**Additional file 1.** Diurnal SB pattern baseline and slope statistics, without controlling for total sedentary time.

**Additional file 2.** Model outputs with controlling for number of morbidities, vs controlling for diabetes, stroke, and arthritis.

## Data Availability

The data that support the findings of this study are available from Dr. Andrea LaCroix on reasonable request in accordance with the WHI (Women’s Health Initiative) publications and presentations policy.

## Abbreviations

BMI: Body mass index
CI: Confidence interval
CVD: Cardiovascular disease
GED: General educational development
MET: Metabolic equivalent of task
MVPA: Moderate-to-vigorous physical activity
OPACH: Objective Physical Activity and Cardiovascular Health
SB: Sedentary behavior
SD: Standard deviation
SE: Standard error
PA: Physical activity
