# Supplementary material for: Diurnal patterns of sedentary behavior and changes in physical function over time among older women: a prospective cohort study
Source: Int J Behav Nutr Phys Act. 2020 Jul 9;17:88. doi: 10.1186/s12966-020-00992-x (PMC7346671; doi:10.1186/s12966-020-00992-x)
Supplement: Supplementary file 1 — Additional file 1. Diurnal SB pattern baseline and slope statistics, without controlling for total sedentary time. [file 12966_2020_992_MOESM1_ESM.docx]

**Additional File 1**: Baseline and slope of physical functioning by diurnal SB pattern and high and low MVPA.


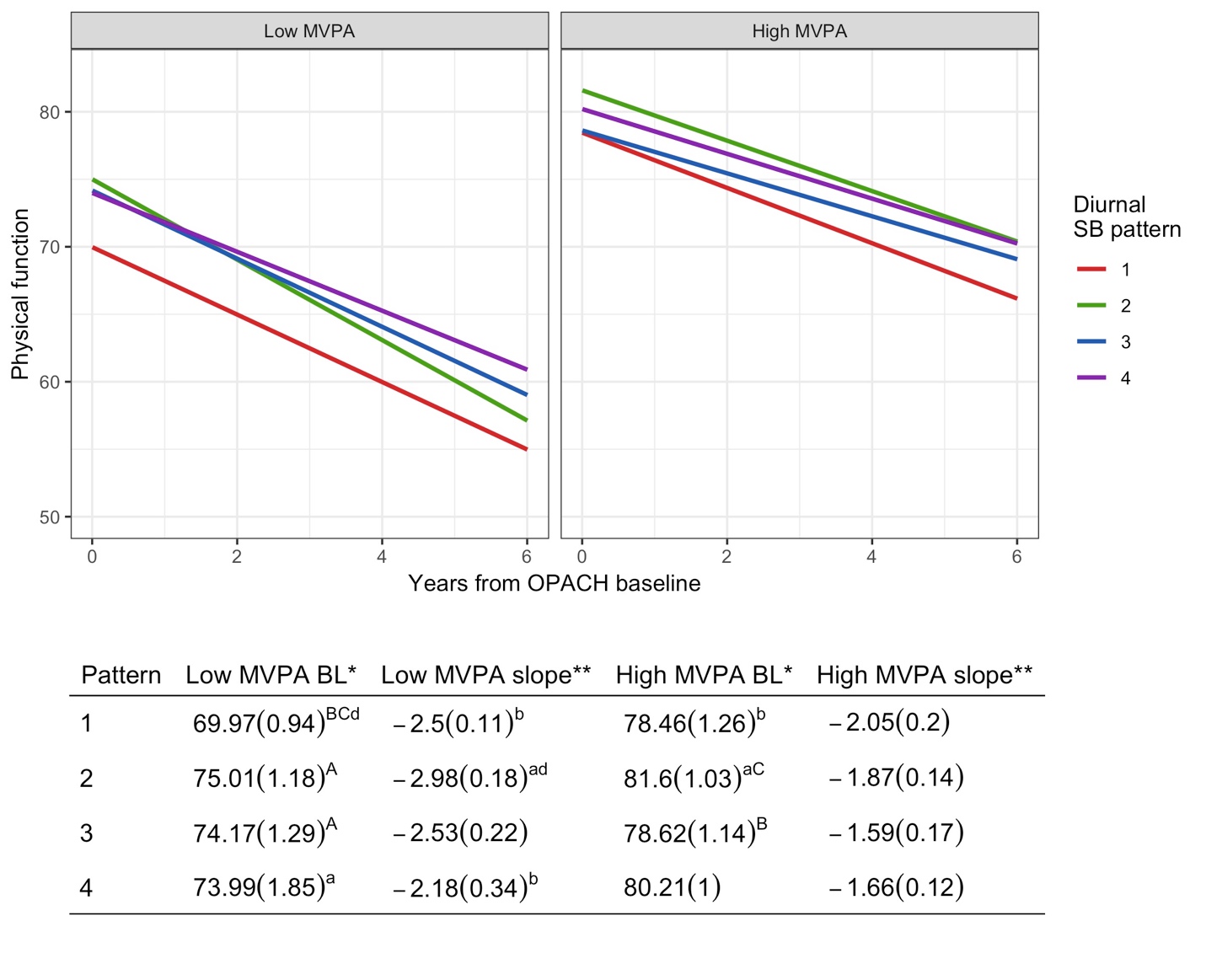


The multivariable model is adjusted for age, race-ethnicity, body mass index (BMI), education, smoking status, alcohol use, number of morbidities, and self-rated health.

BL and slope estimates (standard error) of PF are derived from 3-way interaction model described above.

MVPA is dichotomized into "high" and "low" according to the median value (43.21 min/day).

Abbreviation: BL= baseline; MVPA = moderate-to-vigorous physical activity

*All patterns had significant differences, p<0.01 between their respective high and low MVPA baseline values.

**Patterns 2 and 3 had significant differences, p<0.01, between their respective high and low MVPA slopes.

^abcd^Lowercase a,b,c,d indicate a significant difference, p<0.05, with the BL or slope of pattern 1,2,3,4, respectively.

^ABCD^Uppercase A,B,C,D indicate a significant difference, p<0.01, with the BL or slope of pattern 1,2,3,4, respectively.
