# Supplementary material for: Diurnal patterns of sedentary behavior and changes in physical function over time among older women: a prospective cohort study
Source: Int J Behav Nutr Phys Act. 2020 Jul 9;17:88. doi: 10.1186/s12966-020-00992-x (PMC7346671; doi:10.1186/s12966-020-00992-x)
Supplement: Supplementary file 2 — Additional file 2. Model outputs with controlling for number of morbidities, vs controlling for diabetes, stroke, and arthritis. [file 12966_2020_992_MOESM2_ESM.docx]

**Additional File 2**

Original model with number of morbidities as a covariate:

| Pattern | Low MVPA BL* | Low MVPA slope** | High MVPA BL* | High MVPA slope** |
| --- | --- | --- | --- | --- |
| 1 | 70.62 (0.98)^BC^ | -2.48 (0.11)^b^ | 78.85 (1.28)^b^ | -2.05 (0.2) |
| 2 | 75.06 (1.18)^A^ | -2.98 (0.18)^ad^ | 81.49 (1.03)^ac^ | -1.87 (0.14) |
| 3 | 74.37 (1.29)^A^ | -2.53 (0.22) | 78.58 (1.14)^b^ | -1.59 (0.17) |
| 4 | 73.68 (1.85) | -2.18 (0.34)^b^ | 79.57 (1.03) | -1.66 (0.12) |

New model replacing number of morbidities covariate with diabetes, stroke, and arthritis covariates:

| Pattern | Low MVPA BL* | Low MVPA slope** | High MVPA BL* | High MVPA slope** |
| --- | --- | --- | --- | --- |
| 1 | 72.02 (0.96)^BC^ | -2.48 (0.11)^b^ | 80.68 (1.26)^b^ | -2.05 (0.2) |
| 2 | 76.73 (1.16)^A^ | -2.95 (0.18)^ad^ | 83.49 (1.01)^acd^ | -1.87 (0.14) |
| 3 | 75.8 (1.28)^A^ | -2.48 (0.22) | 80.54 (1.12)^b^ | -1.59 (0.17) |
| 4 | 75.55 (1.86) | -2.19 (0.34)^b^ | 81.48 (1.01)^b^ | -1.66 (0.12) |

*All patterns had significant differences, p<0.01 between their respective high and low MVPA baseline values.

**Patterns 2 and 3 had significant differences, p<0.01, between their respective high and low MVPA slopes.

^abcd^Lowercase a,b,c,d indicate a significant difference, p<0.05, with the BL or slope of pattern 1,2,3,4, respectively.

^ABCD^Lowercase A,B,C,D indicate a significant difference, p<0.01, with the BL or slope of pattern 1,2,3,4, respectively.
